# Epithelial thickness remodeling after small incision lenticule intrastromal keratoplasty in correcting hyperopia measured by RTVue OCT

**DOI:** 10.1186/s12886-023-03272-x

**Published:** 2024-01-08

**Authors:** Yahui Dong, Jie Hou, Jing Zhang, Yulin Lei, Xinghua Yang, Fangfang Sun

**Affiliations:** Jinan Mingshui Eye Hospital, Number 5601, Longquan Road, Zhangqiu District, Jinan, 250200 China

**Keywords:** Cornea, Epithelium, Hyperopia, Lenticule implantation, RTVue OCT

## Abstract

**Purpose:**

To characterize the in vivo corneal epithelial thickness (CET) remodeling profile in a population of eyes after small incision lenticule intrastromal keratoplasty (SMI-LIKE) for hyperopia.

**Methods:**

The CET profile was measured by RTVue-100 Fourier-domain OCT system across the central 6-mm diameter of the cornea of 17 eyes from 12 subjects (five males and seven females) who accepted corneal stromal lens implantation surgery for correcting hyperopia. The CET were measured at positions with a radius of 0–1.0 mm, 1.0–2.5 mm (divided into eight quadrants) and 2.5–3.0 mm (divided into eight quadrants) from the corneal center. Corneal maximum simulated keratometry (Km) was measured by Pentacam HR anterior segment analyzer to analyze CET changes. The examination data of subjects were collected in four time periods, which were preoperative, short-term postoperative (one week after surgery), mid-term postoperative (the last review within 3–6 months after surgery), and long-term postoperative (the last review over 1–2.5 years after surgery). The changes of CET were compared and analyzed in the four time periods.

**Results:**

Mean CET in 0–1.0 mm, 1.0–2.5 mm and 2.5–3.0 mm of the cornea decreased in one week after surgery, respectively, as compared to CET in the preoperative period, which turned from 55.06 ± 0.82 μm、54.42 ± 0.75 μm、53.46 ± 0.60 μm to 51.18 ± 1.05 μm (*P* = 0.005), 49.38 ± 0.70 μm (*P* = 0.000), 51.29 ± 0.59 μm (*P* = 0.025). In the mid-term postoperative period, mean CET in 0–1.0 mm and 1.0–2.5 mm areas kept thinner than mean CET in the preoperative period, CET in 0–1.0 mm is 50.59 ± 0.76 μm (*P* = 0.000),CET in 1.0–2.5 mm is 50.23 ± 0.57 μm (*P* = 0.000), while mean CET in 2.5–3.0 mm area recovered to the same thickness as the preoperative level, which is 54.36 ± 0.66 μm (*P* = 1.000), until the long-term period, CET stabilized in the above doughnut pattern.

**Conclusions:**

After stromal lenticule implantation for hyperopia, CET showed a remodeled form of thinning in the 0–2.5 mm area and thickening in the 2.5–3.0 mm area, and remained stable within one year after surgery.

## Background

Corneal refractive surgery corrects refractive errors by changing the morphology of cornea, the epithelial thickness remodels after refractive surgery, which can mask the presence of an irregular stromal surface from front surface morphology to a certain extent. The general remodeling trend is that the corneal epithelium will try to compensate and recover to the preoperative corneal morphology, so corneal epithelial remodeling is also related to refractive regression, which has been described by Dr. Dan Z. Reinstein's group [1].

RTVue is an ultra-high speed, high-resolution optical coherence tomography (OCT) (Optovue Inc., Fremont, CA, USA). Compared with in vivo confocal microscopy (IVCM) and very high-frequency digital ultrasound (VHF), OCT demonstrates better patient comfort due to its non-contact method, since examinations which contact cornea could compress the epithelium and lead to thinner results [2].

Stromal keratophakia was developed to correct high hyperopia or aphakia [3]. In the early years, the technique was less established, expensive and resulted in poor visual recovery. With the development of femtosecond lasers, stromal lenticules with precise diopter have been studied and used in treating keratectasia [4, 5], hyperopia [6, 7], presbyopia [8], corneal perforation [9], etc. Currently, an extremely accurate stromal lenticule implantation can be achieved.

Dan Z. Reinstein [10] measured the epithelial thickness profile by Artemis 1 (Ultralink LLC) very highfrequency (VHF) digital ultrasound scanning in a population of normal eyes, and demonstrated that the epithelial thickness is characterized by non-uniformity. On an average, the epithelium was thicker inferiorly than superiorly, and thicker nasally than temporally. As described by Alfred Vogt in 1921 [11], the corneal epithelium has the ability to alter its thickness profile to compensate for the changes in stromal surface curvature in order to try and re-establish a smooth, symmetrical optical surface. However, in refractive surgery, it can lead to refractive regression due to filling the ablation zone. In patients with keratoconus [12] and LASIK [13] correction of hyperopia, the corneal epithelium is reshaped like a "doughnut", and in patients with myopia, the central thickness of the corneal epithelium is thickened.

Hence, we described the corneal epithelium remodeling following hyperopic SMI-LIKE with RTVue OCT, and compared it with the previous conclusion in epithelial remodeling following hyperopic LASIK. We also discussed the effect of corneal epithelium remodeling on refractive regression and evaluated the surgical effect in this study.

## Subjects and methods

### Patients

In this retrospective study, 17 eyes of 12 patients (five males and seven females) between July 2019 and August 2021 at the Jinan Mingshui Eye Hospital, China, were enrolled*.* Table 1 shows the demographic data of all subjects. This study was approved by the Ethics Committee of the Jinan Mingshui Eye Hospital Review Board (Ref Ethics/2022/001). The age range of subjects was 11–35 years, with a mean age of 20.67 ± 5.73 years. The inclusion and exclusion criteria were based on Dr. Zhou's research [14], except that we included two teens (11 years old and 16 years old, respectively), who had undergone sequential sessions of perceptual learning for amblyopia [15] in the case of high hyperopia corrected with frame glasses, but had not achieved satisfactory results. They had persistent poor vision with severe visual fatigue, and wished to improve their vision. Each patient (minors with their guardians) provided signed informed consent. The study adhered to the tenets of the Declaration of Helsinki. Spherical equivalent (SE) ranging from 2.25 to 9.50 diopters sphere (DS), with an average of 5.37 ± 1.80DS, median 5.50DS. The cylinder ranged from -0.25 to -3.50 D, with an average of -1.26 ± 0.95D.

**Table 1:** Demographic data of subjects

Subjects	Eyes	Male	Female	Adults (18 ~ 35)	Teens(11 and 16)	Average age(years old)	SE 0 ~ + 3.00D	SE + 3.00D ~ + 6.00D	SE + 6.00D ~ + 9.00D
12	17	5	7	10	2	20.67	2	10	5

### Surgical techniques

All surgeries were performed by the same surgeon (YL). Details of the SMI-LIKE procedures has been described in detail in our previous article [16]. The donor eye matching the degree of the recipient was selected before surgery (astigmatism ≤ 0.25D). Small incision lenticule extraction (SMILE) was performed first for the myopic donor. A femtosecond laser (Zeiss, Germany) was used to make the pocket on the recipient’s eye, the diameter of corneal cap was 7.5 mm and its thickness is 120 μm, and the lens diameter is 6.5 mm. The lens was removed and temporarily stored in the equilibrium solution for use. SMILE operation was performed on the recipient hyperopia first, by which, -0.50D and the recipient’s astigmatism were removed according to the parameters. The diameter of the pocket was 7.5 mm, the length of the incision was 4 mm, the corneal cap thickness was 130 μm, diameter of the lens was 6.5 mm, and the lens was removed. The donor corneal lens was soaked with 0.22% riboflavin for 90 s, then washed with normal saline to remove excess riboflavin, and implanted into the corneal pocket of the recipient’s eye. The center of the implanted lens would be carefully aligned with the visual axis.

### RTVue OCT and pentacam examination

**Fig. 1:**
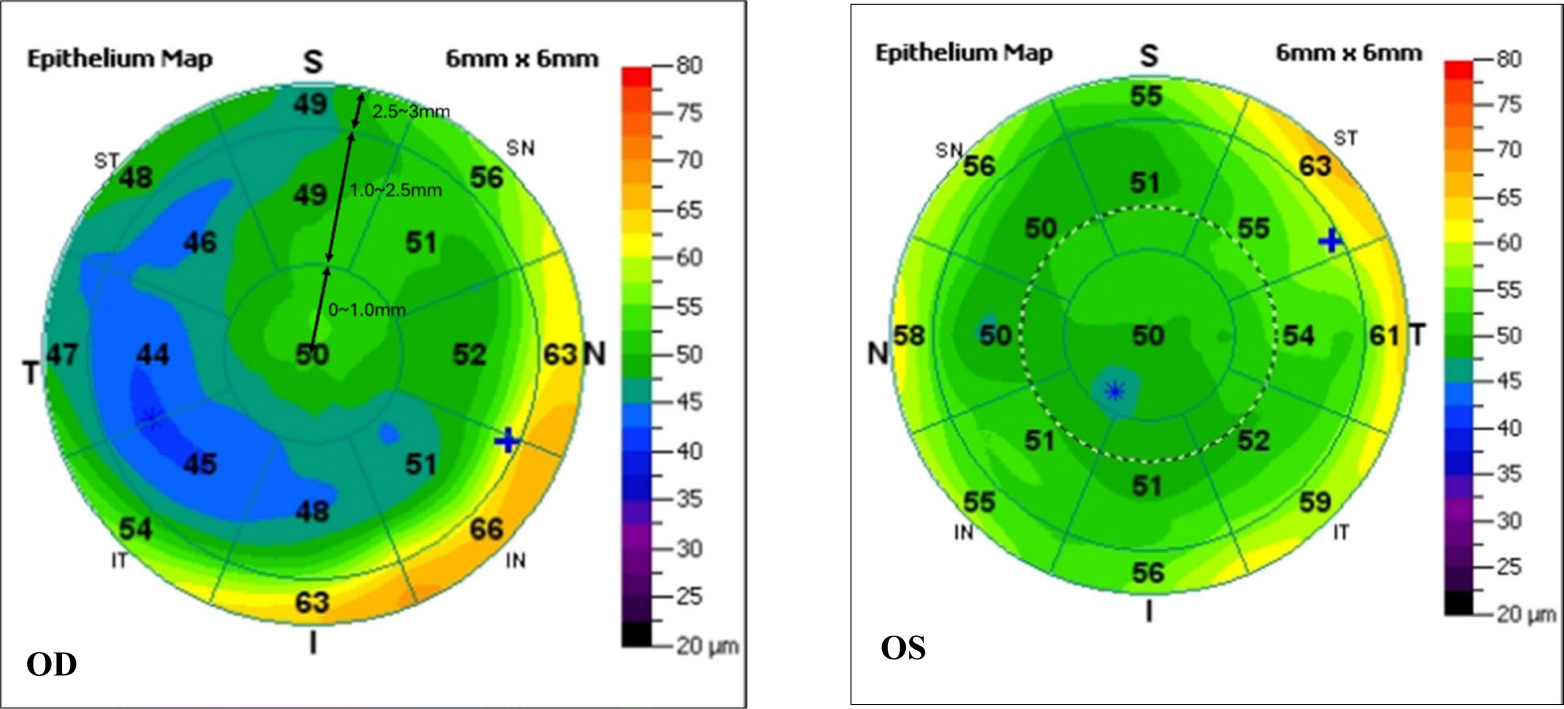
The pachymetry (corneal thickness) map and the CET ranges at the positions with different radii. T, temporal; N, nasal; S, superior; I, inferior

CET was measured by RTVue-100 Fourier-domain OCT system (Optovue Inc., Fremont, CA,USA) and corneal maximum simulated keratometry (K_m_) was measured by Pentacam HR anterior segment analyzer (Oculus, Germany). The two instruments operated in a similar manner. All the operations were completed by trained skilled technicians in the dark room. The subject was seated, with jaw and forehead leaned against the headrest. During the measurement, the subjects opened their eyes as wide as possible and stared at the indicator light spot, and the examinations were completed without blinking. Pentacam operations were completed with quality specifications showing “OK”. OCT scan was automatically processed by RTVue-CAM software (Version 6.11; Optovue Inc.), providing the pachymetry (corneal thickness) map and indicated CT and CET ranges at the positions with radii of 0.0–1.0 mm (central zone), 1.0–2.5 mm (divided into eight quadrants) and 2.5–3.0 mm (divided into eight quadrants) from the corneal center, there were totally 17 regions per cornea (Fig. 1).

### Visual measurements

All subjects underwent uncorrected distance visual acuity (UDVA) and corrected distance visual acuity (CDVA) at 2.5 m acquired by the standardized logarithmic visual acuity chart. The subjects underwent manifest refraction with both normal and dilated pupil preoperatively and manifest refraction with normal pupil postoperatively.

### Statistical analysis

Data analysis was performed using SPSS 26.0 software (International Business Machines, Armonk, NY, USA). Repeated measures analysis of variance was used to compare the CET between four time periods before and after surgery. Comparison between preoperative CET and long-term postoperative CET of different quadrants was performed using paired sample t-test for data conforming to normal distribution, and paired sample rank sum test was used to compare the CET with not normal distribution.

Linear regression analysis was performed between 1) the spherical equivalent refraction of the planted lenses (D) and the central CET with a radius of 0–1.0 mm from the corneal center changes (thinning parts of long-term postoperative CET compared with preoperative CET) (μm); 2) the spherical equivalent refraction of the planted lenses (D) and the maximum simulated keratometry changes between preoperative and long-term postoperative periods (D). The linear regression equation and coefficient of determination (R2) was calculated for each correlation.

The comparison of the maximum simulated keratometry changes between preoperative and long-term postoperative periods in 0 ~ 3D, 3 ~ 6D, 6 ~ 9D subjects was performed using one-way analysis of variance.

Descriptive statistics were performed with Microsoft Excel 2016 (Microsoft Corp, Redmond, Washington). *P* < 0.05 was considered statistically significant.

## Results

### Average CET changes in 0–1.0 mm, 1.0–2.5 mm, and 2.5–3.0 mm areas

Mean CET in 0–1.0 mm, 1.0–2.5 mm and 2.5–3.0 mm of the cornea decreased in one week after surgery, respectively, as compared to CET in the preoperative period, which turned from 55.06 ± 0.82 μm、54.42 ± 0.75 μm、53.46 ± 0.60 μm to 51.18 ± 1.05 μm (*P* = 0.005, R = 93.0%, “R”stands for the rate of change, R = postoperative CET/preoperative CETx100%) 、49.38 ± 0.70 μm (*P* = 0.000, R = 90.7%) 、51.29 ± 0.59 μm (*P* = 0.025, R = 96.5%). In the mid-term postoperative period, mean CET in 0–1.0 mm and 1.0–2.5 mm areas kept thinner than mean CET in the preoperative period, CET in 0–1.0 mm is 50.59 ± 0.76 μm (*P* = 0.000,R = 91.9%),CET in 1.0–2.5 mm is 50.23 ± 0.57 μm (*P* = 0.000,R = 92.3%), while mean CET in 2.5–3.0 mm area recovered to the same thickness as the preoperative level, which is 54.36 ± 0.66 μm (*P* = 1.000,R = 101.7%), until the long-term period, CET stabilized in the above doughnut pattern (Table 2).

**Table 2:** Average CET in 0–1.0 mm, 1.0–2.5 mm, and 2.5–3.0 mm areas (μm)

Range	0–1 mm	1–2.5 mm	2.5–3 mm
Preop	55.06 ± 0.82	54.42 ± 0.75	53.46 ± 0.60
Short-term postop	51.18 ± 1.05^a1^,R = 93.0%	49.38 ± 0.70^b1^,R = 90.7%	51.29 ± 0.59^c1^,R = 95.9%
Mid-term postop	50.59 ± 0.76^a2^,R = 91.9%	50.23 ± 0.57^b2^,R = 92.3%	54.36 ± 0.66^c2^,R = 101.7%
Long-term postop	50.35 ± 0.82^a3^,R = 91.4%	49.81 ± 0.69^b3^,R = 91.5%	53.78 ± 0.91^c3^,R = 100.6%
P(compared with preop)	P^a1^ = 0.005	P^b1^ = 0.000	P^c1^ = 0.025
	P^a2^ = 0.000	P^b2^ = 0.000	P^c2^ = 1.000
	P^a3^ = 0.000	P^b3^ = 0.000	P^c3^ = 1.000

*R* postoperative CET/preoperative CETx100%

**Fig. 2:**
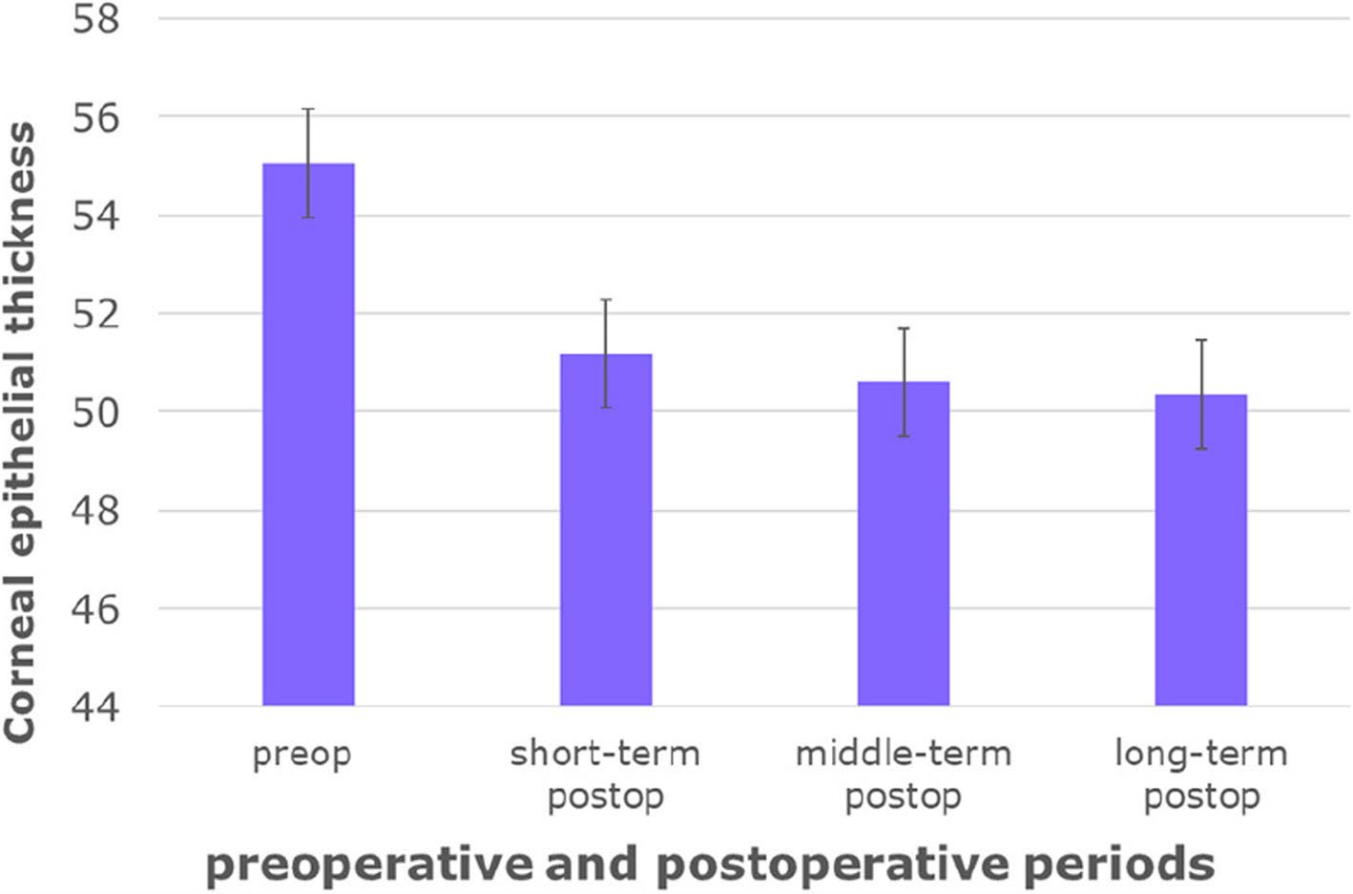
Corneal epithelial thickness distribution in regions with a radius of 0–1.0 mm from the corneal center in different time periods

**Fig. 3:**
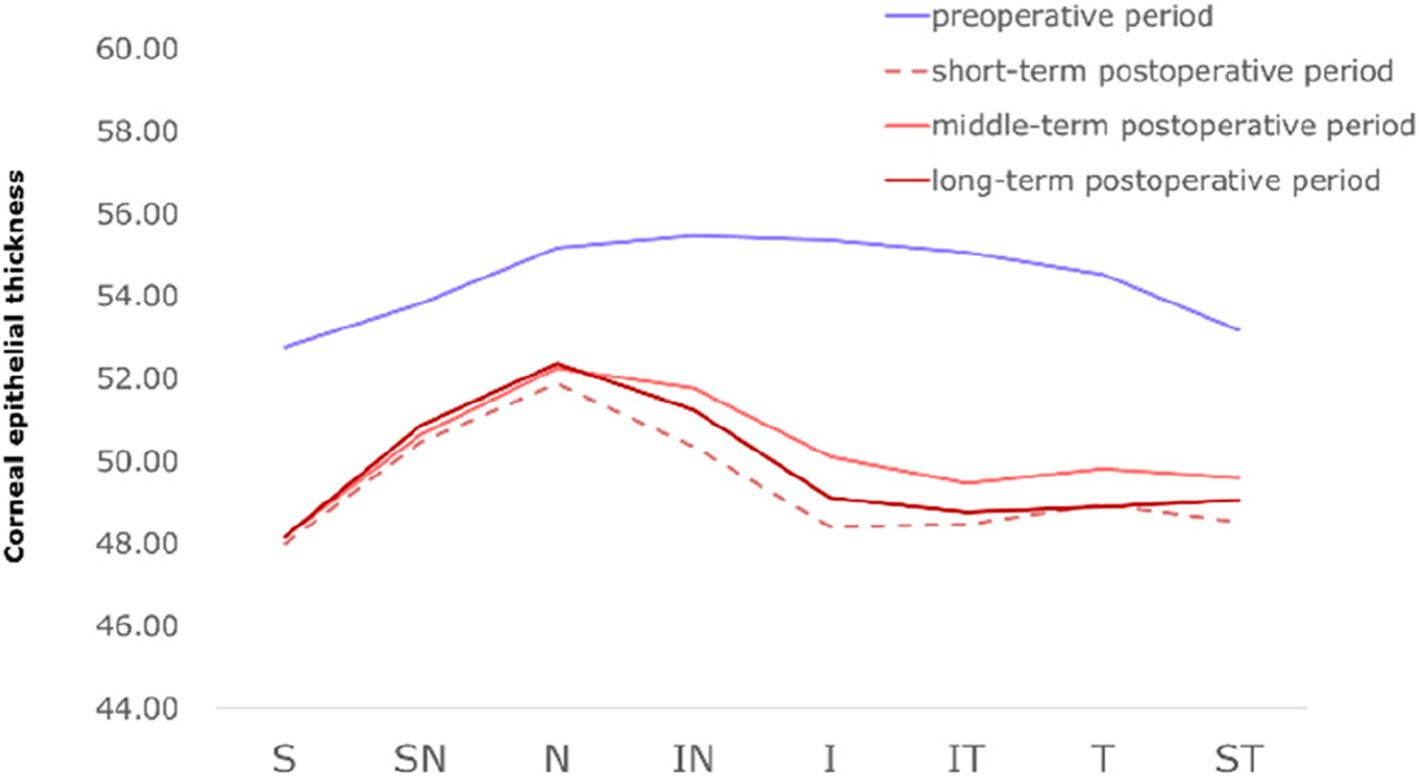
Corneal epithelial thickness distribution in regions with a radius of 1.0–2.5 mm from the corneal center in different time ranges. T, temporal; N, nasal; S, superior; I, inferior

**Fig. 4:**
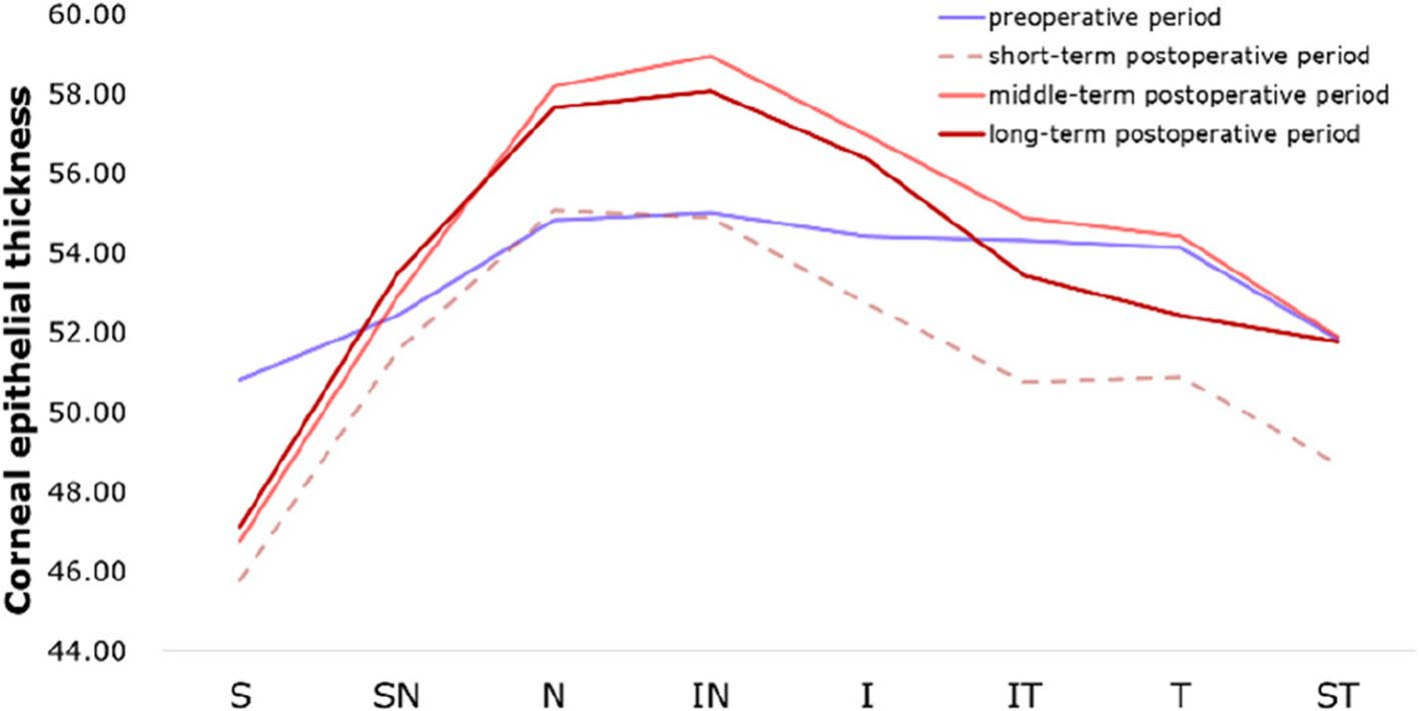
Corneal epithelial thickness distribution in regions with a radius of 2.5–3.0 mm from the corneal center in different time ranges. T, temporal; N, nasal; S, superior; I, inferior

Figures 2, 3, 4 intuitively sketched the epithelial changes in each area, the postoperative CET, from short-term to long-term, in the location with a radius of 0–1 mm (Fig. 2) and 1.0–2.5 mm (Fig. 3) from the corneal center was decreased compared with the preoperative thickness. CET in the zones with a radius of 2.5–3 mm from the corneal center decreased in short-term postoperative period, except CET in N and IN zones which had almost no change. In the mid-term and long-term periods, CET in all zones with a radius of 2.5–3.0 mm from the corneal center became increasingly thicker, and approached or exceeded the level of the preoperative CET (Fig. 4).

### The CET changes in 17 zones between preoperative and long-term postoperative periods.(17 zones include the 0 ~ 1 mm zone, 8 quadrants in 1 ~ 2.5 mm and 8 quadrants in 2.5 ~ 3.0 mm)

The long-term postoperative central CET with a radius of 0–1.0 mm from the corneal center was 50.35 ± 0.82 µm, which indicated significant decrease compared to the preoperative 55.06 ± 0.82 µm (t = 8.025, *P* = 0.000). The CET changes in each quadrant before and long-term (≥ 1 year) after the surgery in 1.0 ~ 2.5 mm, 2.5 ~ 3 mm areas is shown in Tables 3, and 4.

**Table 3:** CET in regions with a radius of 1.0–2.5 mm from the corneal center in preoperative and postoperative hyperopia patients

Time	S (μm)	SN(μm)	N(μm)	IN(μm)	I(μm)	IT(μm)	T(μm)	ST(μm)
Pre	52.76 ± 3.36	53.82 ± 2.96	55.18 ± 3.09	55.47 ± 3.47	55.35 ± 3.41	55.06 ± 3.29	54.53 ± 3.34	53.18 ± 3.34
Post(≥ 1y)	48.18 ± 2.86	50.88 ± 3.16	52.35 ± 3.90	51.24 ± 3.56	49.12 ± 3.00	48.76 ± 3.21	48.88 ± 3.79	49.06 ± 3.60
R(%)	91.3	94.5	94.9	92.4	88.7	88.6	89.6	92.2
P	0.000	0.001	0.002	0.000	0.000	0.000	0.000	0.000

*T* temporal, *N* nasal, *S* superior, *I* inferior, *R* postoperative CET/preoperative CETx100%

**Table 4:** CET in regions with a radius of 2.5–3.0 mm from the corneal center in preoperative and postoperative hyperopia patients

Time	S (μm)	SN(μm)	N(μm)	IN(μm)	I(μm)	IT(μm)	T(μm)	ST(μm)
Pre	50.82 ± 3.09	52.41 ± 2.43	54.82 ± 2.07	55.00 ± 3.18	54.41 ± 3.54	54.29 ± 2.80	54.12 ± 2.78	51.82 ± 2.81
Post(≥ 1y)	47.12 ± 3.69	53.47 ± 4.36	57.65 ± 5.44	58.06 ± 5.56	56.35 ± 4.82	53.41 ± 1.56	52.41 ± 5.82	51.77 ± 4.55
R(%)	92.7	102.0	105.2	105.6	103.6	98.4	96.8	99.9
P	0.000	0.259	0.021	0.016	0.050	0.544	0.238	0.488

*T* temporal, *N* nasal, *S* superior, *I* inferior, *R* postoperative CET/preoperative CETx100%

Long-term postoperative CET in all zones with a radius of 1.0–2.5 mm from the corneal center also decreased significantly, as shown in Table 3. In terms of the radius of 2.5–3.0 mm from the corneal center, only CET in S zone showed decrease, but CET in SN, I, IT, T and ST indicated no significant difference compared with preoperative CET. CET in N and IN increased compared to the preoperative level (Table 4).

### Correlation between the spherical equivalent refraction of the planted lenses and the central epithelial thickness changes

**Fig. 5:**
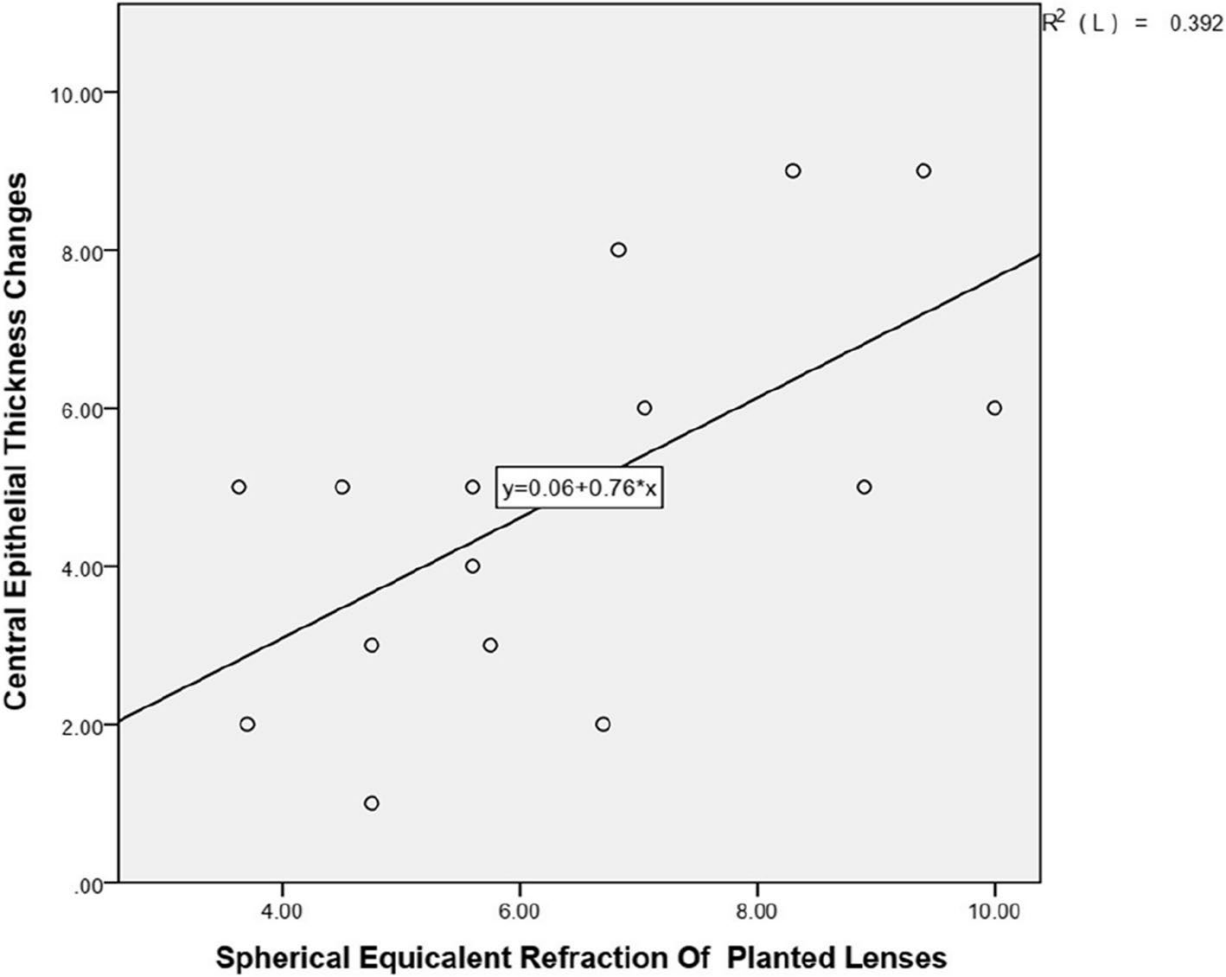
Correlation between the spherical equivalent refraction of the planted lenses (D) and the central CET with a radius of 0–1.0 mm from the corneal center changes (thinning parts of long-term postoperative CET compared with preoperative CET) (μm). One outlier was eliminated in this chart. The linear regression equations and coefficients of determination (R2) are indicated

Figure 5 shows a scatterplot for the spherical equivalent refraction of the planted lenses for the central CET with a radius of 0–1.0 mm from the corneal center changes (thinning parts of long-term postoperative CET compared with preoperative CET) (μm). A statistically significant correlation between the spherical equivalent refraction of the planted lenses and the thinning CET parts in the central zone (R2 = 0.392, *P* = 0.01) was noted. This indicated that the central corneal epithelial thickness was thinner in eyes after higher refractive implantation.

### Correlation between the spherical equivalent refraction of the planted lenses and the maximum simulated keratometry changes

**Fig. 6:**
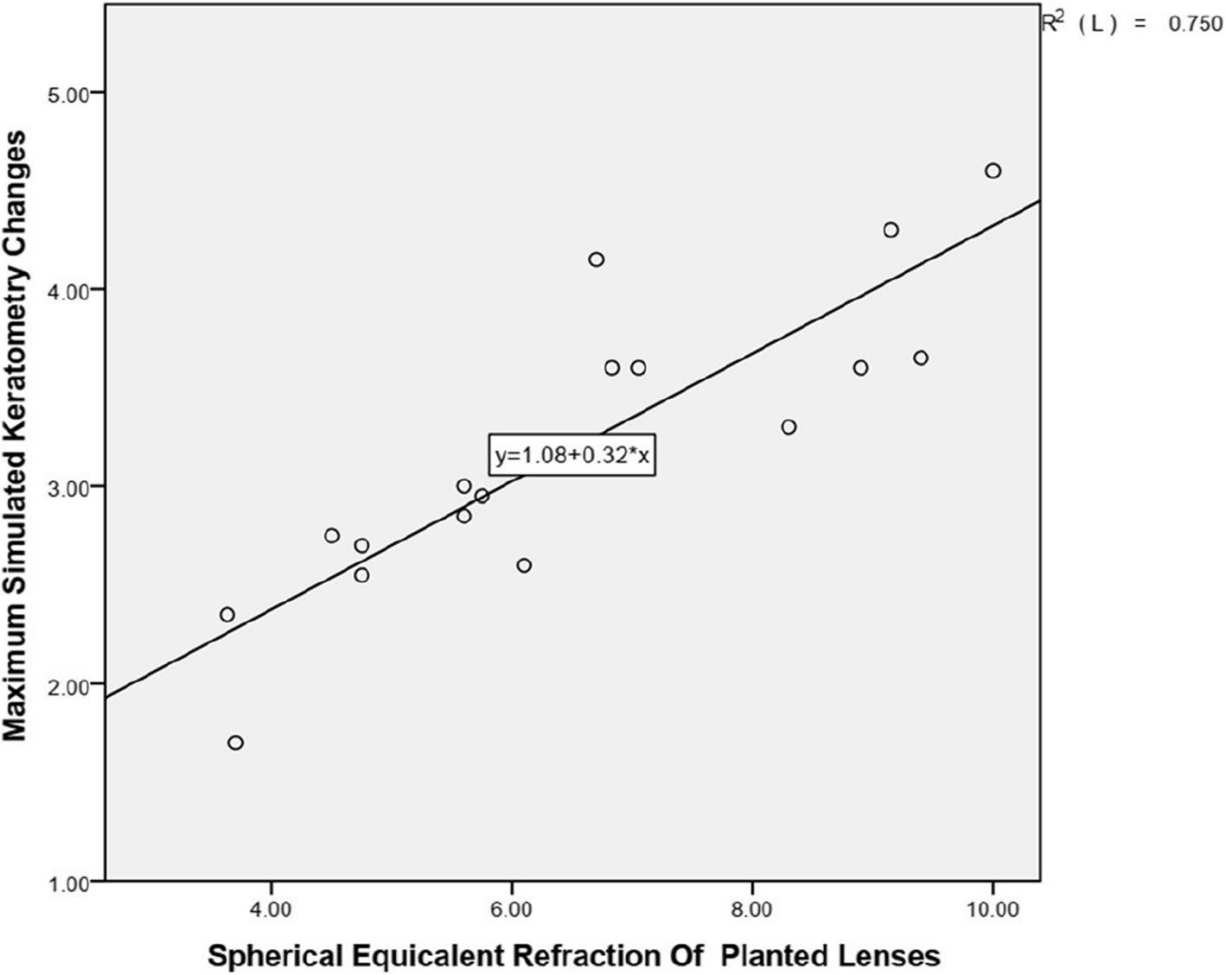
Correlation between the spherical equivalent refraction(SE) of the planted lenses (D) and the maximum simulated keratometry(Km) changes between preoperative and long-term postoperative periods (D). The linear regression equations and coefficients of determination (R2) are indicated

Figure 6 and Table 5 shows the spherical equivalent refraction of the planted lenses and the maximum simulated keratometry (Km) changes between preoperative and long-term postoperative periods. A statistically significant correlation between the spherical equivalent refraction of the planted lenses and the refraction change of the maximum simulated keratometry (R2 = 0.750, *P* = 0.000) was noted. This indicated that the cornea after higher refractive implantation has a steeper maximum simulated keratometry.

**Table 5:** The Km changes between preoperative and long-term postoperative periods corelated with the planted lenses (D)

SE	0 ~ 3.0D	3.0 ~ 6.0D	6.0D ~ 9.0D
n	2	10	5
Km^a^(preop)	42.3 ± 0.42	43.19 ± 1.20	42.34 ± 1.34
Km^b^(Long-term postop)	44.9 ± 0.14	46.16 ± 1.62	46.23 ± 1.85
Km^b−a^	2.55 ± 0.28	2.97 ± 0.68	3.89 ± 0.54
*P* = 0.025, F = 4.827			

Value P,F: Comparison of average value between the Km^b−a^ value of 0 ~ 3D,3 ~ 6D,6 ~ 9D subjects

Km^a^The maximum simulated keratometry in preoperative period

Km^b^The maximum simulated keratometry in long-term postoperative period

Km^b−a^Difference value of the maximum simulated keratometry between postoperative period and preoperative period.

### Visual outcomes after SMI-LIKE

**Fig. 7:**
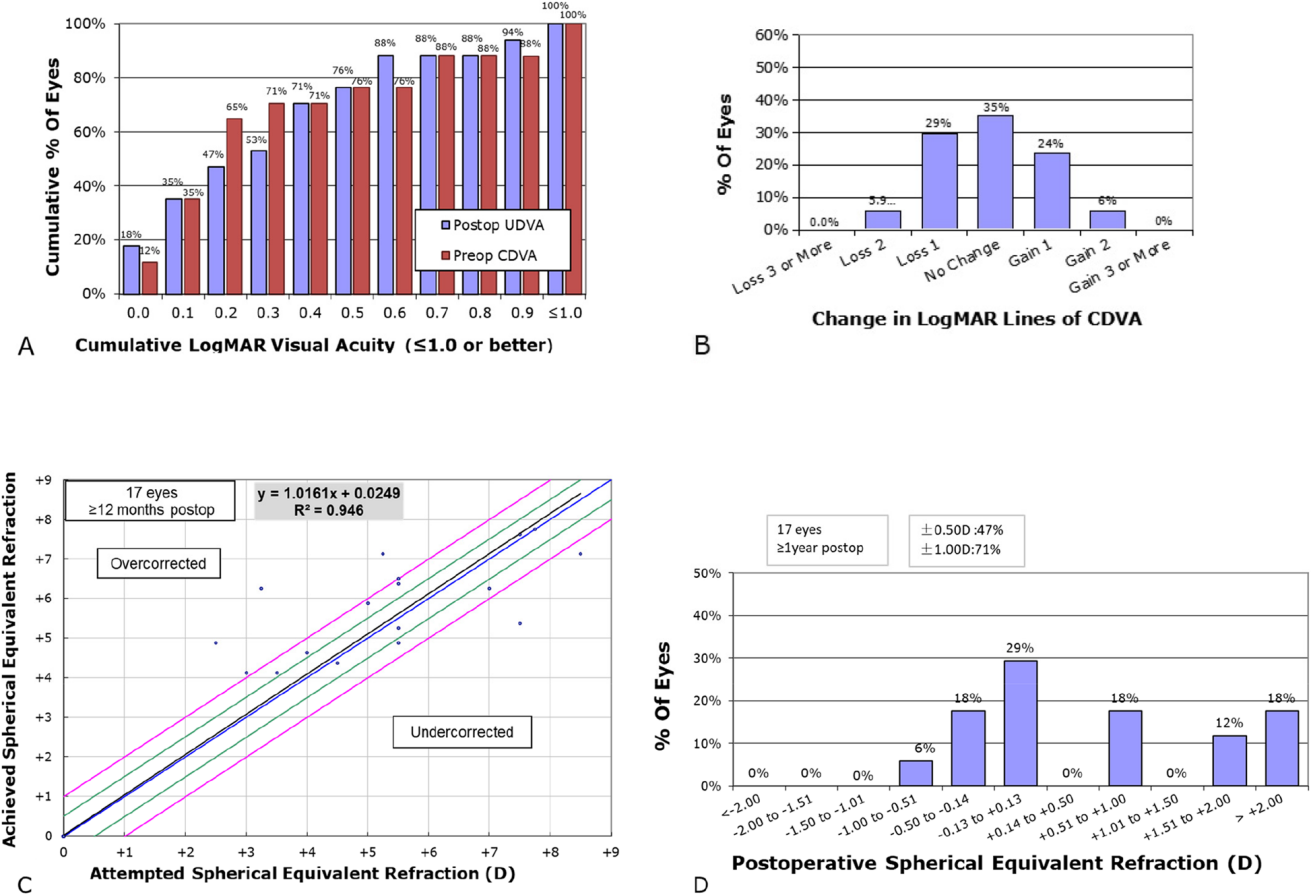
Visual analyses followed small-incision lenticule intrastromal keratoplasty (SMI-LIKE). **A** Postoperative uncorrected distance visual acuity (UDVA). **B** Change in corrected distance visual acuity (CDVA). **C** Scatter plot of attempt versus achieved spherical equivalent (SE) refraction. **D** Postoperative SE distribution

Figure 7A shows the cumulative percentage of UDVA in the last follow-up visit ≥ 1 years postoperatively. A total of 10 eyes (59%) had postoperative UDVA equal to or better than preoperative CDVA. Figure 7B shows the change in the LogMAR lines of CDVA, a total of 11 eyes (65%) had postoperative CDVA equal to or better than preoperative CDVA. Figure 7C shows the scatter plot of the attempted versus achieved SE postoperatively. Patients with low degree tend to be over-corrected while those with high degree tend to be under-corrected, two high degree of hyperopia (+ 7.50D SE and + 9.25D SE) eyes showed a significant undercorrection or refractive regression, the rest is within the forecast. A total of 71% of eyes were within ± 1D in a manifest optometry with normal pupil postoperatively (Fig. 7D).

## Discussion

This is a study to characterize the in vivo CET remodeling over a 6-mm diameter area in eyes after hyperopic SMI-LIKE from one week postoperative to long-term postoperative period more than one year. All eyes showed an epithelial doughnut pattern, in the long-term after surgery, we found an average CET of 50.35 ± 0.82 μm at the 0–1.0 mm area, 49.81 ± 0.69 μm at the 1.0–2.5 mm area and 53.78 ± 0.91 μm at the 2.5–3.0 mm area. Similar to post-LASIK profile, post-SMI-LIKE epithelium also remodeled as a doughnut shape, this is consistent with Zheng Wang, et al.’s conclusion [17]. We wanted to compare it with the post-LASIK profile to explore the long-term effects of different surgical methods for correcting hyperopia, but at our surgical center, patients who chose LASIK to correct hyperopia in recent years were mostly with low to moderate hyperopia accompanied by large astigmatism, so we could not collect relevant data for comparative analysis. Dr. Reinstein’s group studied the epithelial thickness profile by Artemis very high-frequency (VHF) digital ultrasound scanning (ArcScan Inc) over a 10-mm diameter area in 65 eyes of 34 consecutive patients at least three months following hyperopic LASIK with a mean attempted spherical equivalent refraction of + 3.84 ± 1.63 D (range: + 0.75 to + 7.13 D) and a mean attempted cylinder of 1.01 ± 1.04 D (range: 0.00 to 5.00 D), and found the average CET was 39.7 ± 5.6 μm at the central thinnest location and 89.3 ± 14.6 μm at the paracentral thickest location, while the mean postoperative maximum simulated keratometry was 48.10 ± 2.10 D (range: 43.20 to 52.60 D) [13]. In this study, in the long-term postoperative period, the thinnest CET in the central area was 48.18 ± 2.86 μm located in S quadrant of the 1.0–2.5 mm annulus, and the thickest CET was 58.06 ± 5.56 μm located in the IN quadrant of the 2.5–3.0 mm annulus, which showed a flatter doughnut pattern of the epithelium in treatment of larger degrees of hyperopia, regardless of differences in race, sample size and measurement methods. Meanwhile, this uneven CET distribution is consistent with CET distribution in the normal cornea, which may be related to the friction of the eyelid against the epithelium during blinking [10].

In the patient with a + 9.50D lenticule implantation, the maximum thinning of epithelial thickness was located in zone I of the 1–2.5 mm ring, which was 13 μm thinner than before surgery. This change was much smaller than that reported by Dr. Reinstein after LASIK hyperopia, and similar to the epithelial thickening after myopia ablation [13, 18]. This could be explained by the epithelial response according to the corneal morphological changes; hyperopic ablations by LASIK form a steep shape in cornea passively by ablating the peripheral optical zone, the corneal shaping after SMI-LIKE is more gradual and on its own. The epithelium appears to compensate more for a localized stromal irregularity.

Corneal topography is useful in predicting the final keratometry after refractive surgery. Central keratometry steeper than 49 to 50 D after LASIK may be associated with a decrease in quality of vision [19]. In our study, the mean postoperative maximum simulated keratometry was 46.68 ± 1.67 D (range: 43.70 to 49.2 D).

Irrespective of the surgical method, the main concerns are the postoperative effect and when to achieve stability. LASIK following hyperopia usually achieved stability within 1–2 years after surgery [20–22]. It has a good therapeutic effect for low hyperopia, but more frequent worse predictability and a loss of best spectacle-corrected visual acuity (BSCVA) [19, 23, 24] in treating higher degrees of hyperopia. Moreover, surgical pattern and optical zone also play roles in the surgical effect [25, 26].

In the visual outcomes, some patients showed over-corrected, we considered that patients with hyperopia are usually accompanied by strong hyper-regulation, most of them have already adapted to about -2.0D of accommodative myopia, so they presented over-corrected postoperatively. Some patients were reserved some degree of hyperopia in the design of preoperatively surgical parameters, because patients with >  + 8.0D hyperopia were difficult to find enough matching donors, and the two minors were retained some degree considering the increase of the eye axial length during growth, so in Fig. 7D, 18% of total showed > 2.00D SE(+ 2.00D ~  + 3.00D) in a ≥ 1 year follow-up visit. The 11-year-old patient’s eyesight turned from a UDVA1.0, CDVA0.1 preoperatively to UDVA 0.4, CDVA0.2 one year after surgery, and the 16-year-old patient went from UDVA1.2, CDVA1.2 preoperatively to UDVA0.9, CDVA0.9 one year after surgery. Patients might get a better vision with a sequential sessions of perceptual learning for amblyopia postoperatively, but most of them refused that due to lack of time or uncertain efficacy.

According to the current results, there is no significant statistical difference in corneal epithelial thickness between the mid-term and the long-term postoperative periods, indicating that the corneal epithelium can reach a stable state within half a year after surgery. Epithelial remodeling and corneal biomechanics both play significant roles in hyperopic refractive regression [27], and the CET changes following SMI-LIKE suggest that this type of surgery may have lower refractive regression rate and level. Refractive regression is not only related to corneal epithelial remodeling, but also to stroma changes after surgery. Following previous studies, some changes have become predictable, thereby improving the accuracy of surgery. In SMILE surgeries, a 10% refractive sphere overcorrection nomogram is commonly used to obtain accurate outcomes [28]. Alió del Barrio et al. considered that some central corneal stroma expanded due to the biomechanical changes induced by SMILE [29], as the division of the residual bed and the cap might cause expansion of the stroma because they are no longer under tension [30, 31]. Based on this theory, there may have a overcorrection after SMI-LIKE surgery due to the expansion of the corneal stroma, but whether the expansion is partially offset by the compression of the implanted stromal lens worth further investigation.

We plan to collect the data of diopter, curvature, visual acuity and stroma changes in different postoperative periods in the future to discuss the safety and stability of SMI-LIKE correction of hyperopia.

In conclusion, after stromal lenticule implantation for hyperopia, CET showed a remodeled form of thinning in the 0–2.5 mm area and thickening in the 2.5–3.0 mm area, and remsained stable within one year after surgery. The higher the degree, the more prone to gothrough refractive regression or undercorrection, prolonging postoperative corticosteroid drops use may be useful in patients with high hypermetropia to prevent refractive regression.

## Data Availability

The datasets used and/or analysed during the current study available from the corresponding author on reasonable request.
